# The epigenetics of color swatches: histone deacetylation regulates green coloration in Chrysanthemum

**DOI:** 10.1093/plcell/koag297

**Published:** 2026-09-25

**Authors:** Gunjan Sharma, Pei Qin Ng

**Affiliations:** Assistant Features Editor, Plant Physiology, American Society of Plant Biologists; School of Biosciences, University of Birmingham, Edgbaston B15 2TT, United Kingdom; Associate Features Editor, The Plant Cell, American Society of Plant Biologists; Department of Plant Sciences, University of Cambridge, Cambridge CB2 3EA, United Kingdom

Fruits and flowers exhibit a wide variety of vibrant colors, whereas foliar tissues are predominantly green. The role of color in plants extends beyond their aesthetics, as colors in fruits and flowers are important for attracting seed dispersers and pollinators. A balance of chlorophyll and carotenoids, along with other pigments such as anthocyanins, dictates the final color of the flower, fruits and leaves. In leaves, colors are regulated by Golden2-like (GLK) transcription factors, which regulate the expression of enzymes controlling chlorophyll and carotenoid production. Apart from “color swatch” control, the GLK transcription factors are essential in regulating chloroplast development and senescence (Qin et al. 2021). Interestingly, chlorophyll and carotenoid accumulation in fruit is GLK-independent, with other factors such as CAROTENOID CLEAVAGE DIOXYGENASE 4 (CCD4) coordinating color (Amaya et al. 2025; Han et al. 2016). However, the precise mechanisms for color regulation in flowers are largely unknown.

In a recent report in *The Plant Cell*, **Huang, Wen, and colleagues** (2026) have identified a novel GLK-independent module that balances chlorophyll and carotenoid pigments to influence flower color, in a mechanism distinct from leaves and fruits. The authors cleverly used ornamental green chrysanthemum flowers (Anastasia Dark Green [ADG]) as a model for dissecting the pathways for chlorophyll accumulation, with a mutant white Chrysanthemum serving as a control, possessing the lowest total pigment of chlorophyll and carotenoid. Somaclonal variation mutants with a gradient of colors correlated with the levels of chlorophyll and carotenoids suggested that the variable content of these compounds is necessary for flower coloration. Transcriptome analysis between ADG florets vs the white mutant showed no significant differential expression of *CmGLKs*, suggesting that a GLK-independent mechanism controlling pigmentation exists in Chrysanthemum. Among the differentially expressed genes involved in chlorophyll and carotenoid biosynthesis, the authors respectively identified key enzymes for pigment biosynthesis: CmHEMA1 and CmCCD4a. In ADG, *CmHEMA1* expression was higher in ADG flowers, whereas *CmCCD4a* expression was lower compared to the white somaclonal variant. A reduction in *CmHEMA1* and *CmCCD4a* expression by RNAi interference resulted in light green and yellow florets, respectively, suggesting their activity on chlorophyll and carotenoid accumulation directly influences flower color.

A yeast one-hybrid (Y1H) screen using *CmHEMA1* and *CmCCD4a* promoters identified nuclear localized AT-HOOK MOTIF CONTAINING NUCLEAR LOCALIZED (AHL) CmAHL23 and CmAHL8-like (CmAHL8L) proteins, as putative regulators. A dual luciferase activity assay showed that CmAHL23 and CmAHL8L transcriptionally repress their respective targets, thereby affecting chlorophyll and carotenoid accumulation. Inhibition of these transcription factors through RNAi produced the opposite floret color vs inhibiting their targets, suggesting CmAHL23 negatively regulates chlorophyll by repressing *CmHEMA1* expression, while CmAHL8L functions similarly by repressing *CmCCD4a* to promote carotenoid accumulation.

*CmAHL8L-RNAi* and *CmHEMA1-RNAi* lines both exhibited similar light green flower color with reduced chlorophyll accumulation, consolidating earlier transcriptomics analysis findings. The authors showed a decreased expression of *CmHEMA1* and an increase in repressor *CmAHL23* in the *CmAHL8L-RNAi,* suggesting a role for *CmAHL8L* in partially regulating chlorophyll biosynthesis through *CmAHL23*. Y1H, ChIP-qPCRs, and dual luciferase assays all showed enrichment of CmAHL8 at the *CmAHL23* promoter, thus confirming the repression of *CmAHL23*.

To understand how this module might be further regulated, the authors performed a Y2H screen and identified an interaction between CmAHL8L and a histone deacetylase (HDT). *CmHDT1* showed higher expression in the green “ADG” flowers. Further analysis of the activity of CmHDT1 showed increased levels of H3K- and H2K27 acetylation on *CmAHL23* and *CmCCD4a* promoters in *CmAHL8L-RNAi* lines. Collectively, Huang and colleagues' (2026) findings suggest that chlorophyll biosynthesis and carotenoid degradation are epigenetically regulated by the CmAHL8L-CmHDT1 complex which facilitates the removal of active acetylation marks from *CmAHL23* and *CmCCD4a* (Fig. 1).

**Figure 1:**
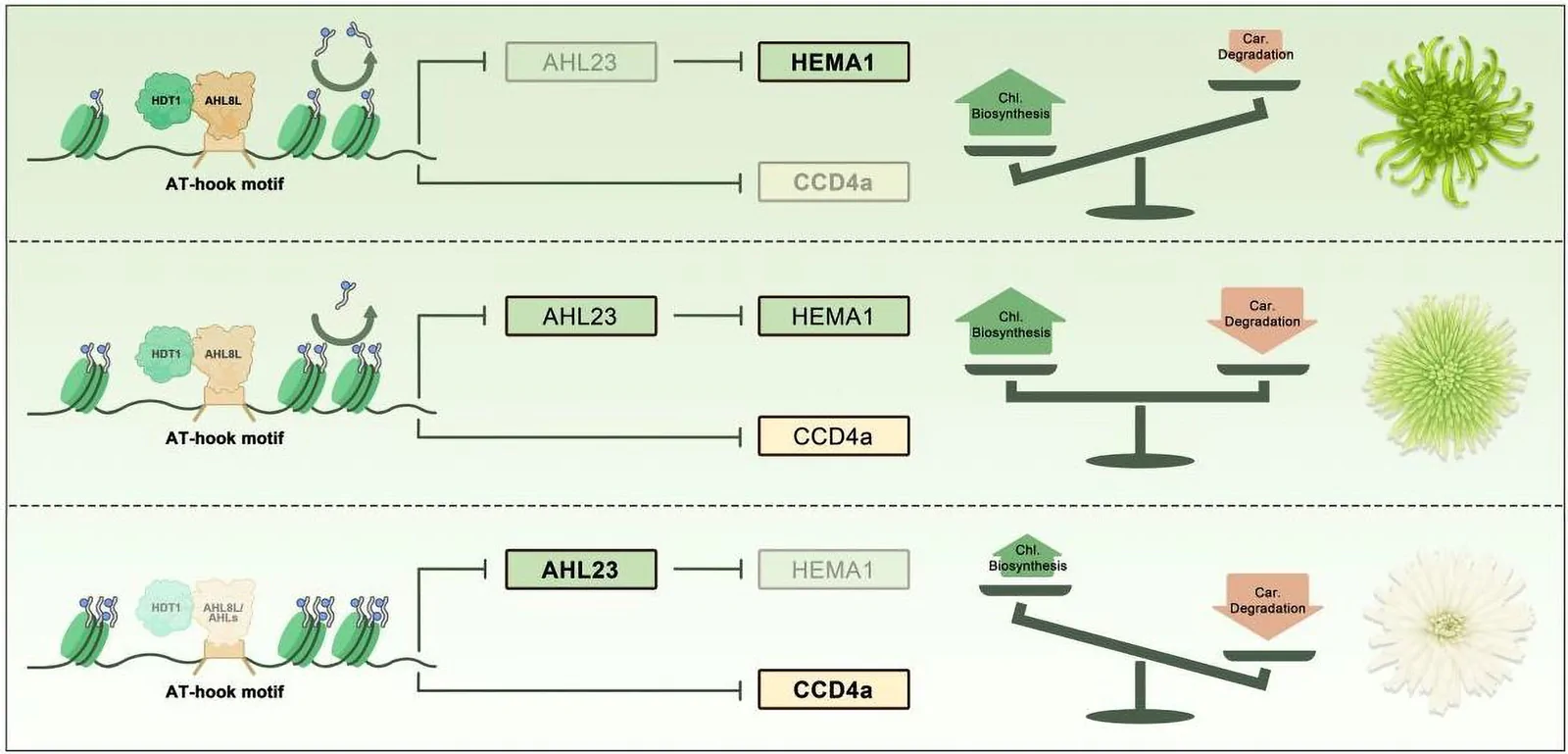
Proposed model of AHL8-mediated regulation of floral pigmentation in flowers. CmAHL8L/CmHDT1 repress Cm*AHL23*, a negative regulator of chlorophyll biosynthesis through *CmHEMA1,* and *CmCCDD4a*, a gene controlling carotenoid degradation (top). Reduced CmAHL8L/CmHDT1 (middle), or complete loss of both CmHDT1 and CmAHL8A (bottom) disrupts this activity, resulting in mixed light green or predominant white coloration (Adapted from Huang et al. (2026)).

In conclusion, Huang et al. (2026) have elucidated a mechanism for “color control” in flowers via the CmAHL8L-HDT1 epigenetic regulatory module, which appears conserved in different flower species such as carnations and lisianthus. The CmAHL8L-HDT1 complex removes transcriptional activating acetylation marks from Cm*AHL23,* depressing the *HEMA1*-dependent chlorophyll biosynthesis while simultaneously inhibiting *CCD4a*-dependent carotenoid degradation to maintain green petals. To that end, restriction of the CmAHL8L-HDT1 complex confers partial or full loss of greening in non-foliar tissue. The authors have provided new insights into the divergence of the molecular mechanisms controlling chlorophyll and carotenoid accumulation in flowers, distinct from leaves. A key challenge in using Chrysanthemum as a model is its hexaploidy. This discovery can help propel the flower industry in color engineering to help balance flower color or produce tailored flowers by manipulating the reported regulators.

## Related articles published in *The Plant Cell*

- Lai et al. (2026) on the role of AT-hook transcription factors in regulating seed development under heat stress in Tartary buckwheat via phase separation.
- Rao et al. (2024) reported on NUDX23 which regulates the level of carotenoids in *Arabidopsis thaliana* at post-translational stages.
- Pei et al. (2025) investigated how DEAR1 transcription factor regulates chlorophyll levels in tomato fruits.

## Data Availability

No new data were generated or analyzed in support of this research.

## References

[koag297-B1] Amaya I et al 2025. Differential expression of CCD4(4B) drives natural variation in fruit carotenoid content in strawberry (Fragaria spp.). Plant Biotechnol J. 23:679–691. 10.1111/pbi.14523. PMC11869172 39840714

[koag297-B2] Han Y et al 2016. Characterization of OfWRKY3, a transcription factor that positively regulates the carotenoid cleavage dioxygenase gene OfCCD4 in *Osmanthus fragrans*. Plant Mol Biol. 91:485–496. 10.1007/s11103-016-0483-6. 27106478

[koag297-B3] Huang H et al 2026. Green flower coloration through AT-hook protein-mediated histone H3K27ac deacetylation in chrysanthemum. Plant Cell. 38:koag225. 10.1093/plcell/koag225. 42525563

[koag297-B4] Lai D et al 2026. Phase separation of an FtAT-hook transcription factor regulates seed development under heat stress in Tartary buckwheat. Plant Cell. 38:koag065. 10.1093/plcell/koag065. 41818704

[koag297-B5] Pei Y et al 2025. The bifunctional transcription factor DEAR1 oppositely regulates chlorophyll biosynthesis and degradation in tomato fruits. Plant Cell. 37:koaf167. 10.1093/plcell/koaf167. 40577586

[koag297-B6] Qin M et al 2021. Genome-wide analysis of the G2-like transcription factor genes and their expression in different senescence stages of tobacco (Nicotiana tabacum L.). Front Genet. 12:626352. https://www.frontiersin.org/journals/genetics/articles/10.3389/fgene.2021.626352/full. 10.3389/fgene.2021.626352 PMC8202009 34135936

[koag297-B7] Rao S et al 2024. Nudix hydrolase 23 post-translationally regulates carotenoid biosynthesis in plants. Plant Cell. 36:1868–1891. 10.1093/plcell/koae030. PMC11653588 38299382

